# Using Smartphone-Based Digital Phenotyping to Predict Relapse in Serious Mental Disorders Among Slum Residents in Dhaka, Bangladesh: Protocol for a Machine Learning Study

**DOI:** 10.2196/79826

**Published:** 2026-02-04

**Authors:** Nadia Alam, Chayon Kumar Das, Neelabja Roy, Domenico Giacco, Swaran P Singh, Sagar Jilka

**Affiliations:** 1 Warwick Medical School, University of Warwick, B 020, Gibbet Hill Road, Coventry, CV4 7AL, United Kingdom, 07300311294; 2 Department of Clinical Psychology, University of Dhaka, Dhaka, Bangladesh; 3 Department of Psychiatry, National Institute of Mental Health and Neurosciences, Bangalore, India; 4 Coventry and Warwickshire Partnership NHS Trust, Coventry, United Kingdom

**Keywords:** mental health, digital phenotyping, passive sensing, low-income, urban settings, mobile phone

## Abstract

**Background:**

Serious mental illnesses (SMIs) are associated with high relapse rates and limited access to continuous care, particularly in low-resource settings such as urban slums. Traditional clinical monitoring is constrained by accessibility and scalability challenges. Digital phenotyping, through passive smartphone data, offers a novel approach to predict relapse by capturing real-world behavioral changes.

**Objective:**

This study aims to evaluate the feasibility and predictive value of smartphone-based digital phenotyping for detecting relapse in individuals with SMIs living in the Korail slum of Dhaka, Bangladesh.

**Methods:**

This prospective 6-month cohort study will recruit 430 participants diagnosed with SMIs who own Android (Google LLC) smartphones. Passive data (eg, screen time, mobility, and call or text frequency) will be continuously collected using a custom-built app (DataDoc). Monthly active data, including symptom and functioning assessments, will be collected via self-report and clinical engagement. Machine learning models will integrate these data to detect early warning signs and predict relapse trajectories.

**Results:**

This study was funded by the NIHR (National Institute for Health and Care Research; award number NIHR200846) in October 2022. Data collection commenced in August 2025 and is ongoing. A total of 14 participants have been recruited, as of January 2026. Preliminary data analysis is ongoing, with expected results to be published in fall 2026.

**Conclusions:**

This study is one of the first to apply smartphone-based digital phenotyping and machine learning for relapse prediction in low- and middle-income countries’ slum settings. The findings will inform scalable, low-cost digital interventions to address the mental health treatment gap in underresourced communities.

## Introduction

### Background

Poor outcomes for patients with serious mental illnesses (SMI) are associated with various factors involving patients, health care providers, and the health care system, in addition to the delivery of inadequate medical services [1-3]. The current clinical ways used to track the progression of SMI may be insufficient or inadequate. Traditionally, health care professionals rely on in-person consultations for assessment and diagnosis [4,5]. However, these clinic-based services come with a variety of logistical difficulties [4], especially for people from impoverished communities [6]. To monitor patients, they must frequently visit a clinical facility, often within restricted operating hours, which can be particularly challenging for individuals dealing with SMI [4]. Additionally, these approaches are demanding in terms of resources because they necessitate one-on-one interactions with a trained health care provider, making widespread adoption difficult. The inherent issues related to accessibility and scalability in these methods create significant obstacles to patient care [7,8].

Remotely collected digital markers may help overcome key challenges in monitoring SMI, such as the reliance on traveling for monitoring and in-person assessments [9,10]. Continuous monitoring of behavioral and physiological data using personal devices has the potential to aid in the identification of personalized indicators for the initiation of illness [4], subsequently enabling the development of tailored treatment strategies [9,11,12]. Smartphones can provide a solution.

### Digital Phenotyping (DP)

Smartphones can be used to collect data to understand mental health status. This technique, called digital phenotyping (DP) [13], is already in use in some behavioral problems and mental disorders, such as posttraumatic stress disorder (PTSD) [14], and SMI such as schizophrenia [15-17] and mood disorders [18-20] to predict relapse, symptom exacerbation, or mood fluctuations [21].

Digital phenotypes are collected either actively or passively [5,22]. Active data collection requires the individual’s active participation through active engagement [23]. This would include self-reported thoughts and symptoms through surveys or scales, whereas passive data collection does not require an individual to perform any specific action outside their regular activities [24]. Passively collected data, such as screen behavior, including screen time, locking or unlocking events, and screen lighting has been shown to reflect mental health states in adults, offering insights into phases of agitation, depression, and variations in sleep quality and patterns [10,23,24]. Accelerometer data from smartphones has also been linked to mental health concerns such as depression and anxiety due to reduced activity, such as mobility and traveling, while audio data, such as read-aloud recordings, help indicate depressive states [23,25]. Within DP, sleep-related features, such as sleep duration, variability, latency, and nighttime phone use, are increasingly recognized as important phenotypes that can reflect underlying mental states. Disturbed sleep, as captured through these DPs, has been linked to elevated stress and SMI [26]. In bipolar disorder, disruptions in sleep patterns can precipitate manic or depressive episodes and persist even during remission, affecting overall functioning [27-29]. Major depressive disorder (MDD) often features insomnia or hypersomnia, with sleep issues potentially preceding depressive episodes and exacerbating their severity [30]. In schizophrenia, sleep abnormalities frequently emerge before psychotic symptoms and are linked to more severe clinical outcomes [30]. Geolocation data offer insights into individuals’ mobility patterns and daily routines [31]. In schizophrenia, reduced GPS-derived mobility, such as spending more time at home and traveling shorter distances, is associated with increased severity of negative symptoms such as social withdrawal and diminished motivation [32]. Similarly, in bipolar disorder, fluctuations in geolocation patterns can reflect mood episodes, with decreased movement often observed during depressive phases [33].

DP has gained significant traction in high-income countries (HICs), where studies have demonstrated its utility in detecting early warning signs (EWS), monitoring symptom fluctuations, and predicting relapse in individuals with SMIs [22,34-37]. For example, in a US-based study, passive sensing data, including GPS location, accelerometer activity, screen use, and call or text logs, was used to identify EWS of psychotic relapse. Machine learning (ML) techniques were applied to detect behavioral anomalies preceding relapse events, demonstrating the potential of DPs for relapse prediction in schizophrenia [22]. A narrative review demonstrated how various digital features, such as geolocation, communication patterns, and activity levels, have been effectively used in schizophrenia research to understand behavioral changes, and studies included were conducted in HICs [16]. In mood disorders, passive data from smartphones and wearable devices, such as GPS location, accelerometer activity, sleep pattern data, and heart rate, were used to estimate depressive and anxiety symptom severity [18,19].

Together, these passively collected DP, such as sleep, mobility, screen use, and activity patterns, offer a continuous, unobtrusive window into individuals’ lived experiences. Their integration into mental health monitoring holds promise for early detection of symptom changes, relapse prediction, and timelier, personalized interventions in the care of individuals with SMI.

### Relapse

Relapse is a significant concern in the management of SMIs. Relapse can lead to adverse outcomes, including poor psychosocial functioning, increased caregiver burden, and higher health care costs [38-40]. Therefore, continuous monitoring and adherence to treatment are crucial in mitigating the risk of relapse and improving long-term outcomes for individuals with SMIs. Most patients go through a period with changes in behavior, which precedes their psychotic relapse, commonly known as EWS [41]. EWS includes changes in sleep patterns, hallucinations, delusions, hostile behavior, cognitive decline, depression, and paranoia [42]. Developing systems that can identify and monitor these EWS could help clinicians intervene early [41]. In a study, anomalies in passive data, such as geolocation, accelerometer readings, and screen state, were 2.12 times more frequent in the month preceding a relapse compared to nonrelapse periods [40,43]. In another study with participants with schizophrenia, the analysis revealed that anomalies in passive data (geolocation, accelerometer data, and screen state) were significantly more frequent before a relapse [43]. Importantly, models incorporating passive data outperformed those relying solely on active survey data in predicting relapse [43]. These findings show the potential of passive smartphone data to serve as early digital markers of relapse, offering a scalable and nonintrusive method for the timely intervention and continuous monitoring in individuals with SMI.

### Low- and Middle-Income Countries (LMICs)

As described above, most of the DP research has been conducted in HICs, raising concerns about generalizability to low- and middle-income countries (LMICs) [44], where digital usage patterns, social determinants of health, and mental health care infrastructure differ substantially [42]. This motivates the need to evaluate whether DP approaches can be adapted and applied effectively in LMIC settings such as Bangladesh, where the burden of untreated SMIs is high and innovative, low-cost monitoring solutions are urgently needed [45,46].

Mental and behavioral disorders account for 12% of the global disease burden, with over 70% of this impact affecting LMICs [47]. In Bangladesh, the treatment gap (ie, the gap between people who need care and the people who obtain care) is over 92% [47], which means that less than 1 in 10 people get the mental health care they need, and the gap is more evident in slums [6,45,47]. People living in slum communities have high rates of SMIs, limited access to mental health services, and conditions of chronic hardship [48]. Therefore, a low-cost, easy-to-use solution to support the identification and monitoring of mental disorders is needed, and DP may provide a solution. DP has paved its way in mental health research [9-11], but most of the research conducted has been in high-income or developed countries; these do not account for findings in LMIC settings. Yet, 80% of people with mental disorders live in LMICs [42]. Social factors such as sudden changes in life, urbanization, and poverty often lead to a high burden of mental illness in several LMICs [42,49-51]. Additionally, there is a significant lack of awareness regarding mental health conditions among LMICs [49]. Slum dwellers here have a high burden of mental health conditions, which remains unnoticed due to a lack of help-seeking or lack of resources [48,49]. The addition of DP will enhance the detection of mental disorders among these communities with little to no cost, as the use of smart devices such as smartphones is common among slum dwellers in Dhaka.

### Aims and Objectives

#### Overview

The proposed study aims to determine whether DPs can effectively predict relapse among residents from the Korail Slum, Dhaka. One of the key outputs of this study will be to provide evidence of the feasibility and reliability of using DP in LMIC settings, particularly slum settings, for more effective mental health monitoring and intervention [47].

#### Primary Objective

We aimed to develop and validate predictive models using smartphone-based DP data to detect and forecast relapse in individuals with serious mental disorders living in the Korail Slum.

#### Secondary Objectives

For secondary objectives, our aims were:

- To explore associations between DPs and clinical facets such as mood states, social withdrawal or functioning, sleep disturbances, and activity levels.
- To assess the feasibility, acceptability, and reliability of smartphone-based DP among slum residents in a low-resource setting.
- To identify contextual and user-level factors (eg, socioeconomic, environmental, and technological) that influence data completeness and engagement with DP tools.

## Methods

### Outcome: Relapse

The primary outcome for prediction modeling will be the occurrence of a relapse event within a 6-month follow-up period defined using prespecified, quantitative, diagnosis-specific criteria based on validated clinical scales, supplemented by structured clinician adjudication. For participants with psychotic disorders, relapse will be defined as either (1) a ≥25% increase from baseline on the Positive and Negative Syndrome Scale (PANSS), sustained for at least 7 days, or (2) deterioration in global functioning as indicated by a decline of ≥10 points on the Global Assessment of Functioning (GAF), confirmed by clinician rating. These thresholds align with relapse definitions used in prior studies in psychosis, where relapse was operationalized using scale-based symptom worsening and functional decline [17,22,43]. For participants with nonpsychotic disorders, including MDD and PTSD, relapse will be defined as clinically significant symptom worsening on the Brief Symptom Inventory (BSI), operationalized as a ≥30% increase from baseline on the relevant symptom subscale and/or meeting caseness on the BSI Global Severity Index (GSI), defined as a total GSI T-score ≥63 together with an increase of at least 7 points from the mean of the 2 preceding assessment scores, indicating a move into or further into the clinically significant distress range [52]. In repeated-measurement and longitudinal studies involving patients with mood, anxiety, and trauma-related disorders, changes in BSI symptom subscales and the GSI have been widely used to capture clinically meaningful worsening over time [52-55]. In these populations, sustained increases from an individual’s own baseline, rather than isolated score fluctuations, are commonly interpreted as reflecting true symptom deterioration. To reduce the risk of misclassifying temporary or situational symptom changes as relapse, clinically meaningful worsening is typically operationalized using relative change thresholds or standardized deviations from baseline sustained across multiple assessments.

Across all diagnostic groups, any psychiatric hospital admission or emergency psychiatric presentation during follow-up will be classified as a relapse event. All algorithm-identified relapse events will undergo blind clinician adjudication by an independent clinician using only symptom scale trajectories and clinical information; the clinician will be blinded to all DP features. Clinician judgment may confirm or refute events meeting quantitative thresholds but will not introduce relapse events in the absence of predefined criteria, thereby preventing post hoc outcome redefinition. Each relapse will be timestamped at the earliest point at which the criteria are met, enabling prediction within predefined risk horizons (eg, relapse within 30 d).

### Study Design

This study is a 6-month prospective cohort study designed to explore the feasibility and reliability of identifying DP to predict relapse of SMIs among slum residents in Dhaka, Bangladesh. This study is part of the broader NIHR (National Institute for Health and Care Research) initiative, Transforming Access to Care for Serious Mental Disorders in Slums (TRANSFORM project) [47], which provides the overarching framework. The TRANSFORM project is dedicated to improving access to mental health care for individuals with SMIs residing in urban slum environments [47]. The TRANSFORM study aims to bridge the treatment gap by developing, implementing, and assessing innovative community-based interventions [47].

### Setting

Participants will be recruited from the Korail slum, one of the largest and most densely populated slums in Dhaka [47]. It is situated between 2 affluent areas and is home to an estimated 200,000 people living in densely populated conditions with limited access to basic services [47,51,56,57]. The community consists of a mix of long-term residents and recent migrants from rural areas, and many inhabitants face precarious employment, housing instability, and health inequities [56,57]. Mental health needs are high, yet access to formal mental health services remains scarce [47].

### Participants and Recruitment

Participants will be selected using purposive sampling. Participants will be identified through the TRANSFORM project [47], where community engagement representatives are well known and respected in the area. A community-based approach will be used to identify and enroll participants, leveraging existing partnerships with local stakeholders. We collaborated with community health workers, nongovernmental organizations, and TRANSFORM [47] community representatives, who had well-established relationships with the residents. These trusted individuals facilitated introductions and provided culturally appropriate explanations of this study to potential participants. Importantly, recruitment will not be limited to individuals already enrolled in TRANSFORM. Community representatives will help identify potential participants, introduce this study, provide culturally appropriate explanations, and facilitate trust with potential participants. Participants will progress through clearly defined stages: approached and screened for eligibility, enrolled if inclusion criteria are met, followed up monthly with both active and passive data collection, and retained in the analytic sample provided sufficient data completeness is achieved. Numbers at each stage will be reported to ensure transparency.

This study will recruit participants diagnosed with SMIs, including MDD, psychotic disorders (such as schizophrenia and bipolar disorder), and PTSD, as per the *DSM-5* (*Diagnostic and Statistical Manual of Mental Disorders, Fifth Edition*) [58]. These conditions were selected due to their high burden in low-resource settings, their recurrent nature, and the critical need for early detection of symptom exacerbation [42,59-61]. Inclusion criteria require participants to be at least 18 years of age, residents of the Korail slum, diagnosed with SMI, and in possession of an Android smartphone. Exclusion criteria include those younger than 18 years of age, those without a smartphone, and individuals diagnosed with substance-induced psychosis or mental illness.

### Sample Size

#### Total Enrollment

Based on findings from the National Mental Health Survey (2019), ≈17% of adults in Bangladesh are estimated to experience a mental health disorder [62]. While specific prevalence rates of SMIs in Korail, Dhaka, are not available, this national estimate provides a useful contextual baseline. Population estimates for Korail vary widely, from 50,000 to 2,00,000 individuals, but for planning purposes, a conservative midpoint estimate of 100,000 residents was used [47,51,57].

To determine an appropriate sample size for estimating a proportion with maximum variability, the standard formula with finite population correction was applied [63-65]:


$$\text{Sample size}=\frac{\frac{z^{2}\times p(1-p)}{e^{2}}}{1+\left(\frac{z^{2}\times p(1-p)}{e^{2}N}\right)}$$

Where Z=1.96 (for a 95% confidence level), *P*=0.5 (maximum variability), e=0.05 (margin of error), and n=100,000 (estimated Korail population). Substituting these parameters yields n of ≈384.16. To account for potential nonresponse or incomplete data, a 90% response rate was assumed:


$$\begin{array}{rl} n_{\text{adjusted}} & =384.16/0.90-426.84\\ n_{\text{final}} & =426.84\approx 430 \end{array}$$

#### Prediction Model Adequacy (Relapse Events)

To power the prediction model, we planned recruitment of 430 participants and assumed a 15% attrition rate (N is ≈366) over 6 months. The primary justification for the sample size is based on the widely accepted events per predictor (EPP) effective degree of freedom (EDF) principle for prediction model development, which mandates a minimum of 10 events per effective predictor.

Based on previous literature, relapse incidences range between 25%‐40% for psychosis [66,67], 15%‐30% for MDD [68], and 10%‐25% for PTSD [69]. Using these rates, we estimated the expected number of relapse events for our planned cohort. To account for diagnostic heterogeneity within the sample, we used existing diagnostic compositions from the TRANSFORM study [70]: (1) 45% psychosis/42% MDD/20% PTSD) − expected ≈ 66‐121 relapse events across the incidence ranges, (2) psychosis: 0.40×366 × 0.25‐0.40=37‐59 events, (3) MDD: 0.40×366 × 0.15‐0.30=22‐44 events, and (4) PTSD: 0.20×366 × 0.10‐0.25=7‐18 events.

Concerning anticipated events (E), after accounting for a 15% attrition rate and the calculated relapse incidence ranges (66‐121 events), the minimum number of events (E) is 66 relapses.

Of the required EDF, given a minimum of E=66, the maximum number of effective predictors (EDF) that the model can reliably support while maintaining the EPP>10 [71] ratio is 6.6 predictors.

About model strategy alignment, our study involves high-dimensional data (numerous digital phenotypes). To ensure compliance with the estimated EDF (≈6‐7 predictors), the modeling pipeline will reduce the large number of raw features into a smaller set of effective predictors through penalized modeling (eg, LASSO [least absolute shrinkage and selection operator], ridge regression, or regularized tree-based methods) and dimensionality reduction or feature aggregation techniques.

### Procedure

Participants will be informed about this study through participant information sheets (PIS) and will be given 48 hours to provide written informed consent. The PIS will be provided in Bengali, the local language, and will clearly explain the purpose of this study, the type of data to be collected, what participation involves, risks and benefits, data security, and the right to withdraw at any time without affecting access to services. Participants will be contacted through community engagement representatives and ongoing recruitment activities under the TRANSFORM project [47]. These representatives, who have established relationships within the community, will distribute the PIS and introduce this study to potential participants. Trained research assistants will obtain written informed consent at the local field office with a witness present from participants willing to take part in this study. This will be done in a private setting to ensure confidentiality, and the information sheet will be explained in Bengali. After obtaining consent, the DataDoc app will be installed on participants’ smartphones to passively collect data. The research team will assist them in downloading the app, granting required permissions (eg, access to device usage), setting up the app to ensure proper functionality, and troubleshooting any technical issues. Participants will be trained on app installation and basic use. Monthly clinical assessments will be conducted either in-person or via telephone, while passive data will be captured continuously in the background. Technical support will be available through community fieldworkers to address issues such as app functioning, battery management, or data sync.

Participants will install DataDoc on their smartphones for data collection as outlined below. This data will be collected via the app automatically and passively once the user gives the app permissions. A link to the app will be sent to participants via email or text (based on their preference). This is an .apk file which they can install on their Android phone. We will support participants to do this (eg, in person in the Korail field office) if needed. Upon downloading the app, they will be asked to give the app permission to access their device usage. Please note, the app also has the potential to connect to music and health data, for example, music data (ie, permission to access their Spotify [Spotify AB] data) and health data (ie, permission to access their Google Health app data), but we will only be using app usage for this study.

Relapse events will be identified using a combined approach. First, thresholds on validated scales (active data) will trigger a potential relapse flag. These events will then be reviewed by 2 independent clinicians and a researcher with expertise in psychiatry and psychology. The researcher will be blinded to the sensor-based DP data and will adjudicate relapse status using only clinical and scale-based information. In case of disagreement, a third senior clinician will review the case to reach a consensus.

### DataDoc App

**Figure 1.**
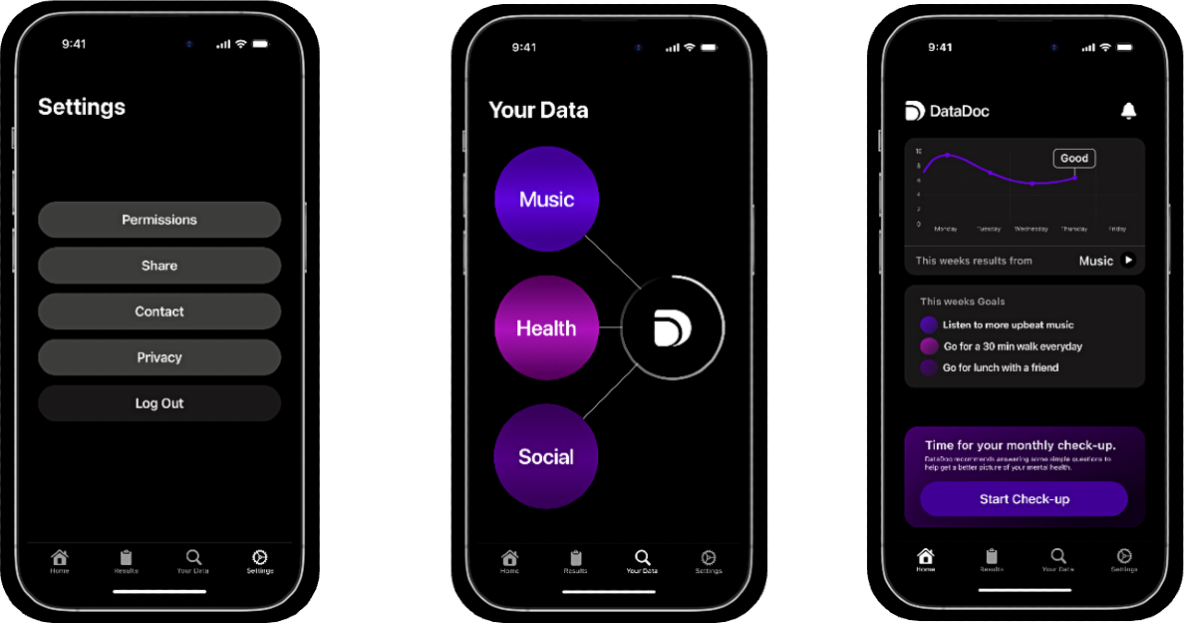
The DataDoc app.

We have developed an app, DataDoc (Figure 1), to collect DP data, which we will use for data collection in this study. Some active data will be collected directly through the DataDoc app, while assessments requiring clinician input will be obtained separately. After giving permissions, the app will sync this data, and then the participant will be presented with a screen asking them to complete the questionnaires outlined below in the active data section. Participants will be asked to complete the questionnaire via the app only. The questionnaire must be completed within 2 weeks of syncing the device, music, and health data. Participants will be called with reminders if they have not completed the questionnaire after 1 week and again after 2 weeks. At this point, we will ask them to resync their data and complete the questionnaire, so all their data is synced to the same time point.

### Metrics

Participants will complete a set of baseline assessments and assessments at intervals as shown in Table 1.

**Table 1.** Timeline of data collection.

Domain, subdomains, and tools			Assessment time points							
			Baseline	Month 1	Month 2	Month 3	Month 4	Month 5	Month 6	Continuously
Active data										
	Sociodemographic profile		✓							
	Clinical profile and history									
		Diagnosis	✓							
		Timeline of diagnosis	✓							
	Clinical assessments									
		Global Assessment of Functioning (GAF)	✓	✓	✓	✓	✓	✓	✓	
		WHOQOL (World Health Organization Quality of Life)	✓	✓	✓	✓	✓	✓	✓	
		Brief Symptom Inventory (BSI) or the Positive and Negative Syndrome Scale (PANSS)	✓	✓	✓	✓	✓	✓	✓	
		Perceived Stress Scale	✓	✓	✓	✓	✓	✓	✓	
		Sleep questionnaire	✓	✓	✓	✓	✓	✓	✓	
	Thought patterns									
		Journaling (optional)	✓	✓	✓	✓	✓	✓	✓	
Passive data										
	Device interactions									
		Phone usage								✓
		Application usage								✓
		Screen time								✓
	Digital interaction									
		Calls (frequency)								✓
		Duration of calls								✓
		Messages (frequency)								✓
	Activities (if applicable)									
		Activities app (sleep and health)								✓
		Sleep								✓
	Movement and location									
		Geolocation (longitude and latitude)								✓
		Duration at location								✓
		Movement time								✓

#### Passive Data

Passive data will be collected continuously, on an event-by-event basis, and will include:

- Device interactions: this study will collect data on how a participant interacts with their digital devices. This includes phone usage, application usage (application history such as application on the foreground, number of application notifications, when an application is installed and removed), and screen time.
- Digital interaction: calls and messaging habits (number of incoming and outgoing messages and calls), frequencies of responses (duration of call; the content of communications will not be accessed or stored).
- Activities (if applicable): sleep and health application installed or removed, and total sleep duration (integrated within DataDoc app).
- Movement and location: geolocation (longitude and latitude), duration at one location, and movement time.

#### Active Data

##### Demographic Questionnaire

Each participant will be provided with a demographic questionnaire containing questions regarding age, gender, level of education, occupation, diagnosis profile, and home address.

Active data will consist of the following and will be collected monthly.

##### Clinical Data

The clinical data consisted of the following:

- GAF is a clinician-rated scale to assess global functioning. GAF represents a latent continuous measure score from 1 (lowest score representing worst symptomology) to 100 (high score representing optimal level of functioning) [72]. This data will be collected from the ongoing TRANSFORM Project [47].
- WHOQOL-BREF (World Health Organization Quality of Life-Brief) is a 26-item self-reported scale to assess quality of life, where scores range from 16 to 112, and a higher score represents a higher quality of life [73].
- BSI is a 53-item self-reported instrument on a 5-point scale used to evaluate psychological symptoms over the past week in patients, nonpatients, and participants involved in experimental research [74]. The BSI consists of 9 main symptom categories: somatization, obsessive-compulsive tendencies, sensitivity in relationships, feelings of depression, anxiety, hostility, phobic anxiety, paranoid thoughts, and tendencies toward psychosis, and higher scores represent more severe symptoms [74].
- The PANSS is a scale used for measuring symptom severity with 3 components of positive, negative, and general psychopathology scales [75]. The total PANSS score is the sum of the scores of the 3 scales, ranging from a minimum of 30 to a maximum of 210, with higher scores indicating more severe symptoms [75].
- Perceived Stress Scale [76] to assess perception of stress with scores ranging from 0 to 40, where higher scores represent higher perceived stress.
- The Nottingham Onset Schedule is a structured interview tool designed to assess the onset of psychosis, and it provides a systematic method of gathering detailed information about the initial presentation and progression of symptoms [77].

##### Activities

The activities consisted of the following:

- Self-reported sleep patterns through a dedicated general questionnaire for this study derived from the sleep questionnaire for adults (part two) by the National Health Service, which will explore the time of the participant going to bed, hours of sleep, number of times the participant woke up, perceived quality of sleep, etc [78].
- For journaling, participants will be asked to “journal,” that is, write down their thoughts and feelings if they wish [79].

### Data Privacy and Security

**Figure 2.**
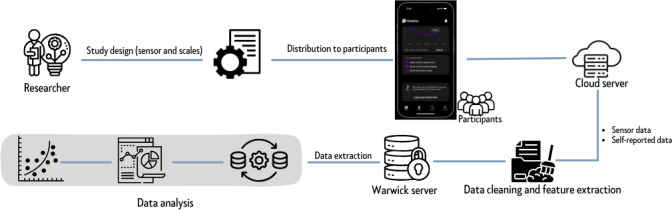
Data flow.

As illustrated in Figure 2, data is first collected on the participant’s phone using the DataDoc app. Data are then securely uploaded to a General Data Protection Regulation–compliant cloud server, where they are stored in encrypted form at rest. From there, encrypted anonymized data are transferred to the University of Warwick’s secure server for long-term storage and analysis, in line with Warwick’s 10-year research data policy. No personally identifiable information (eg, phone numbers and message content) is collected at any stage. Access to both the cloud server and Warwick’s secure server is restricted to authorized study personnel using password-protected accounts.

### Analysis

#### Overview

This study adopts a multimodal ML-driven analytical framework to integrate and analyze both active and passive data streams collected via the DataDoc app. The goal is to develop predictive models for relapse detection and to explore associations between specific digital phenotypes and clinical dimensions. The analysis pipeline will involve preprocessing of the collected active and passive data, feature engineering, labeling (when needed), ML model selection, model fitting, and evaluating the predictive validity of our multimodal data, followed by further interpretation and deployment when needed.

#### Analysis of Active Data

##### Data Preprocessing

###### Cleaning and Standardization

We will remove any inconsistencies and standardize the format of the active data to ensure uniformity. For demographic questionnaire variables such as gender, occupation, or diagnosis, any typographical errors will be corrected, and if any absurd numerical value is recorded for age or education, it will be flagged for replacement. For clinical assessment instruments, it must be ensured that item responses are within the appropriate range of the corresponding scales. If not, the out-of-range and/or strange entries will be flagged for further systematic treatments.

###### Handling Missing Values

Missing responses will be addressed through multiple imputation techniques or median replacement where feasible, and guided by manuals as necessary. For demographic variables, if there are randomly missing variables, then mean or median imputation for numerical variables and mode for categorical variables may be considered. In cases of substantial missingness, a separate category such as “unknown” may be assigned. A threshold for what constitutes “substantial” will be prespecified during analysis planning. For the clinical assessment scales, if there is item-level missingness, then the person’s mean imputation will be used. If there is any block-level missingness that affects entire subscales, then the participant would be flagged and dropped if needed.

###### Encoding Categorical Variables

Categorical variables, such as demographic data like gender, will be converted into numerical representations suitable for computational steps and ML models. Variables such as education level can be mapped to ascending integers, and nominal ones such as occupation can be encoded with 1-hot or label encoding.

### Feature Engineering

#### Extraction of Relevant Features

We will identify and extract features from active data that are indicative of behavioral and psychological factors associated with relapse. Demographic variables such as occupation or education level can be grouped into a factor such as socioeconomic status if deemed necessary for future analysis. From the responses on the clinical assessment instruments such as GAF and others, scoring will be done as per their respective manuals after ensuring all responses are in their appropriate ranges. Additionally, for scales such as WHOQOL-BREF, where specific items such as items 3 and 4 are also reverse-worded, they will be reverse-scored before further processing. Its domains, such as physical health, psychological health, social relationships, and environment, will also have their domain scores from averaging across items of each domain. Similarly, for BSI, subscale scores will be computed for all the symptom dimensions as well as the global indices. For PANSS, subscores of domains (positive, negative, and general psychopathology) will be computed from summing items of each domain, while total PANSS will be the sum of all items. For a scale such as Nottingham Onset Schedule, where key outputs are dates such as that of the onset of prodrome or psychosis, the date-field variables will be converted to the computational datetime format, following which durations such as that of untreated psychosis will be computed comparing as intervals between symptom onset and treatment start dates or likewise.

#### Symptom Severity Trends

Longitudinal changes in clinical assessments (eg, PANSS and BSI scores) will be modeled to track deterioration or improvement.

#### Sentiment and Thematic Analysis

Journaling entries will undergo natural language processing (NLP)–based sentiment and thematic analysis to extract emotional patterns. Given the linguistic context of Dhaka slum residents, entries are often written in a mixture of Bengali and romanized Bengali (“Banglish”), and off-the-shelf pretrained models are limited for such code-mixed and colloquial data. We therefore will adopt a staged approach: (1) start with strong multilingual transformers (mBERT and XLM-R); (2) fine-tune Bengali models (BanglaBERT [80] or IndicBERT [81]) on a small, study-specific, annotated code-mixed corpus; and (3) add light normalization (transliteration handling; domain lexicon for common Banglish expressions). Multilingual transformers (especially XLM-R) generally outperform vanilla mBERT on low-resource languages, and Bengali-specific models (BanglaBERT and IndicBERT) provide monolingual priors we can adapt via task-specific fine-tuning. To ensure validity, we will manually label a stratified subsample (≈10%‐20% of entries) for sentiment or topic, compute interannotator agreement, and use this “gold” to calibrate and evaluate the NLP models; NLP features will be treated as exploratory covariates rather than primary end points. Recent work has released Bengali-English code-mixed sentiment datasets (eg, BnSentMix) [82] and shows that fine-tuning or further pretraining on code-mixed data substantially improves over vanilla multilingual models, which supports our feasibility plan.

### Analysis of Passive Data

#### Overview

High-dimensional smartphone sensor features (eg, mobility, sleep, social communication, and screen-use variability) will be condensed into a limited number of aggregated, clinically interpretable composite variables before model training. Analyses will use penalized regression and regularized tree-based algorithms (eg, gradient boosting) to mitigate overfitting.

#### Data Preprocessing

##### Noise Reduction

Continuous, high-frequency passive raw data, as captured from the digital sensors, are often noisy, containing irrelevant signals that need to be smoothed out for more reliable analysis and modeling. Therefore, artifacts such as random or closely spaced idle screen event sessions can be merged, and spatial smoothing and clustering can be applied over GPS jitters. Zero-duration, irrelevant calls or text communications will be filtered out. Kalman filters or moving averages will be used for smoothing out sensor spikes for motion data.

##### Outlier Detection

Tailored to the behavioral context in question, anomaly detection techniques for variables from such passive data sources will be used. To start, the fast and easily interpretable methods, such as IQR-based filtering, will be applied to handle and assess the quality of individual behavioral variables such as screen time, call counts, and their durations, duration of app use or of movement, and of movement distance as well. For more multivariate, time-varying, and longitudinal contexts, techniques of isolation forests and rolling *z* scores will identify and address deviations and outliers from the standard range and/or baseline.

##### Normalization

We will normalize passive data to ensure consistent scales across different features. To maintain comparability across different devices and participants, minimum-maximum scaling or *z* score normalization will be applied to passive data features.

### Feature Engineering

#### Feature Extraction

We will be extracting relevant information from continuous passive data streams.

#### ML Steps

In this study, predictive modeling using ML will focus on leveraging the DPs collected through participants’ smartphones as the primary features (predictors), with relapse as the primary target outcome. Clinical symptom scores (eg, PANSS, BSI, and WHOQOL-BREF) will serve as secondary outcomes for exploratory analyses and for validating relapse definitions. By analyzing patterns in the DPs, such as device interactions, sleep patterns, movement, and app usage, the model will learn to identify subtle behavioral changes that may be indicative of mental health shifts, such as the onset of relapse. By training the model on historical data from participants, where mental health conditions have been assessed via standardized clinical questionnaires, the system will aim to forecast future mental health status or relapse risk with high accuracy. This approach will enable the integration of both passive smartphone data and active clinical assessments to provide a comprehensive and early-warning prediction system for serious mental disorders in low-income settings.

### Primary Analysis

#### Overview

For the ML analysis, we define the prediction horizon as the risk of relapse within the next 30 days. This choice is informed by prior DP research demonstrating elevated behavioral anomalies in the 2 weeks preceding relapse and increased anomaly frequency within a 30-day window surrounding relapse [17,83]. Features from passive and active smartphone data will be derived using a 14-day look-back window before the relapse index date. To reduce bias from the clustering of events, a 30-day washout period after each relapse will be applied, during which no new relapse event will be coded.

The primary supervised modeling approach follows a structured analytical pipeline, beginning with temporal feature aggregation, model training, and validation. Passive DP features, including screen usage, mobility, and sleep-related indicators, will be aggregated across predefined sliding windows (eg, last 1, 7, and 30 d) to condense high-frequency time series data into summary statistics. This aggregation facilitates noise reduction, captures short-term behavioral trends, and converts raw sensor streams into fixed-length feature vectors suitable for supervised learning.

As aggregation may introduce feature redundancy and multicollinearity, dimensionality reduction and feature selection techniques, including principal component analysis, singular value decomposition, regularized LASSO regression, mutual information filtering, and SHAP (Shapley Additive Explanations)-based feature importance assessment, will be applied to retain the most informative behavioral features while preserving interpretability. Interaction features will also be derived to examine nonlinear relationships between digital phenotypes (eg, movement variability and screen usage), enabling representation of higher-level behavioral constructs such as mobility, rest, activity, social behavior, and device use. These derived components will be mapped to labeled relapse and nonrelapse windows and aligned with static demographic or clinical covariates, when applicable.

All primary models will be trained with relapse as the target outcome. To ensure robust evaluation and prevent temporal and subject-level data leakage, the dataset will be divided into time-aware training (70%‐80%) and testing (20%‐30%) partitions, ensuring that future behavioral data are not used to predict earlier outcomes. Model selection and hyperparameter tuning will be conducted using nested cross-validation within training folds. Temporal validation procedures, including rolling window and expanding window validation, will be used to assess model stability over time. Nested cross-validation will also be used to minimize overfitting and optimistic bias.

Given the relatively low frequency of relapse events, class imbalance will be addressed using case-control sampling by time (matching nonrelapse windows from the same observation period) and cost-sensitive learning approaches, with higher penalties assigned to misclassified relapse events. Synthetic oversampling techniques, such as the synthetic minority oversampling technique, will not be applied due to the risk of temporal leakage in longitudinal data.

#### Primary Model Specific and Evaluation

The prespecified primary supervised model will be gradient boosting (XGBoost or LightGBM), selected a priori for its robust performance with multimodal behavioral data and demonstrated effectiveness in prior DP studies of relapse prediction [7,17]. The gradient boosting model, together with the feature construction and validation strategy described above, constitutes the single prespecified primary modeling pipeline for this study. Model performance will be internally validated using temporal cross-validation and bootstrap optimism correction, with reporting of discrimination (area under the receiver operating characteristic curve) and calibration metrics (calibration slope and Brier score). At a prespecified probability threshold, sensitivity, specificity, positive predictive value, and negative predictive value will also be reported for relapse prediction within the 30-day horizon. Calibration quality will be further assessed using expected calibration error, and decision curve analysis will be conducted to evaluate potential clinical utility across a range of risk thresholds. Explainability methods, including SHAP values and permutation importance, will be applied to identify the most influential digital phenotypes contributing to relapse risk.

### Secondary and Exploratory Analyses

#### Alternative Supervised Models

As sensitivity analyses, the performance of the primary gradient boosting model will be benchmarked against regularized logistic regression and random forest models. These models provide interpretable and clinically acceptable baselines and will be trained and evaluated using the same feature set, outcome definition, and validation framework as the primary analysis.

#### Unsupervised and Deep Learning Approaches

As exploratory analyses, unsupervised anomaly detection methods (eg, autoencoders and clustering approaches) and time-series deep learning models (eg, long short-term memory and transformer-based architectures) will be examined to explore temporal behavioral patterns and relapse trajectories. These analyses will be contingent on data volume and relapse event frequency and are intended for hypothesis generation and pattern discovery rather than primary inference.

#### Exploratory Use of Active Data and Data Fusion

Analyses using clinical symptom scores (eg, PANSS, BSI, and WHOQOL-BREF) will be conducted as secondary exploratory models to contextualize relapse prediction and support interpretation of behavioral features. To maximize predictive insight, multimodal fusion approaches will be explored at the feature and decision levels. Feature-level fusion will integrate passive DP data with active clinical assessments, while decision-level fusion will involve combining outputs from multiple models using ensemble techniques such as stacking and boosting. Multimodal fusion and ensemble methods will be used strictly as secondary analyses to complement, but not replace, the prespecified primary supervised model [7,17,83].

### Ethical Considerations

This study was approved by the Biomedical and Scientific Research Ethics Committee at the University of Warwick (reference number Biomedical and Scientific Research Ethics Committee 98/23‐24). Data collected from participants are securely stored and will not be available for sharing due to privacy considerations. The smartphone app (DataDoc) used for data collection and the feature processing code are available upon request for research purposes.

Participants were informed about the nature, procedures, potential risks, benefits, and voluntary nature of the research before enrollment. Informed consent was obtained from all individual participants before data collection. Participants received detailed information sheets and provided written consent confirming their voluntary participation and understanding of their rights, including the right to withdraw at any time without penalty. The consent process ensured comprehension of this study’s aims, data collection procedures, privacy protections, and how data would be used and stored.

To recognize participants’ time and engagement in this study, compensation was provided at a rate of 1200 BDT per month for the duration of their involvement. This compensation was disclosed to participants during the consent process and was approved as part of the ethics review.

All data collected from participants are securely stored in accordance with local and international data protection standards and are not publicly available due to privacy and confidentiality considerations. Identifiable personal information has been protected and anonymized where possible to prevent unintended disclosure.

## Results

This study received funding from the UK NIHR (award number NIHR200846) under the TRANSFORM Project [47]. Pilot data collection began in August 2025 in the Korail slum, Dhaka, and as of October 2025, recruitment is ongoing. Data analysis, including feasibility and predictive modeling, commenced in December 2025, with preliminary findings anticipated in mid-2026 and expected results to be published in fall 2026. A total of 14 participants have been recruited, as of January 2026.

## Discussion

### Principal Findings

The results of this study will offer critical insights into the application of DP for predicting the relapse of SMIs in low-resource settings, specifically among slum residents in Dhaka, Bangladesh. By using passive data collected from participants’ smartphones, such as device interactions, sleep patterns, movement, and social engagement, this study aims to assess the ability of ML models to detect early signs of relapse. Our findings will contribute to the broader understanding of how DP can be applied in real-world settings to improve mental health care, especially in LMICs where access to traditional mental health services is difficult or limited [42,49].

This study’s innovative approach of combining passive data with clinical assessments allows for the development of personalized, dynamic interventions tailored to individuals’ behavioral patterns. This could represent a significant advance over static mental health monitoring methods, offering more timely and effective interventions. However, several challenges and limitations must be acknowledged. First, while DP offers a novel way to monitor behavioral changes, the accuracy and validity of these markers in predicting clinical outcomes require further exploration. The reliance on self-reported clinical assessments may introduce bias [4], as participants’ perceptions of their mental health may not always align with objective symptoms. Moreover, such assessments often lack ecological validity, as they are typically conducted in clinical settings and may not capture the nuances of daily life. In contrast, DP offers the potential to monitor individuals in real-world contexts, providing richer insights into behavior and symptom fluctuations outside the clinic [12,13]. Furthermore, privacy concerns surrounding the continuous collection of personal data must be addressed [12], even though strict data protection protocols are in place.

The results will also have implications beyond the scope of this study. The potential for DP biomarkers to serve as dynamic tailoring variables in adaptive interventions could reshape how mental health treatments are delivered, particularly in resource-poor settings. As smartphones become increasingly prevalent in LMICs, leveraging these devices for mental health care could provide a scalable, cost-effective solution that reaches underserved populations. The findings from this study will help guide future research and intervention strategies, paving the way for larger, more comprehensive studies in similar settings.

### Limitations

A key limitation of this study is the requirement for participants to own an Android smartphone, which may systematically exclude individuals with SMIs who are older, poorer, or have lower educational attainment. These subgroups may be among those most vulnerable to relapse, meaning our sample could underrepresent the most at-risk individuals [84,85]. To mitigate bias, we will document sociodemographic and clinical characteristics of enrolled participants and compare these with available data on the broader Korail slum population. In addition, subgroup analyses stratified by socioeconomic and demographic variables will be conducted to evaluate whether predictive performance varies across groups. These steps will help us interpret findings in light of potential exclusion and inform future efforts to reach harder-to-engage individuals with SMIs.

Finally, our sample size estimation was based on the national prevalence of mental disorders in Bangladesh (17%). We acknowledge that this may not accurately capture the epidemiology of SMI in slum settings, where prevalence may be higher or differently distributed due to socioeconomic disadvantage, overcrowding, and limited access to care. As such, our study may be under- or overpowered relative to the true prevalence in Korail. This limitation will be transparently acknowledged in reporting and highlights the need for more robust epidemiological studies of SMI within urban slum populations.

Several practical limitations may affect feasibility. First, requiring participants to install the app as an .apk file may present challenges for individuals with low technical literacy. To address this, in-person installation support and illustrated step-by-step instructions in Bengali will be provided. Second, continuous passive data collection, particularly from GPS sensors, has the potential to increase battery consumption. Given that there is potentially unstable access to electricity in slum environments, participants may disable or uninstall the app to conserve power. While the DataDoc app has been optimized for low-frequency sampling to reduce power demand, battery drain remains a possible barrier to adherence. Furthermore, to minimize battery drainage in a setting with intermittent electricity, the app will upload data only when connected to Wi-Fi or charging [86]. Third, concerns about data privacy and security may limit participant engagement. Although strict protocols are in place, including anonymization and secure server storage, participants may still perceive privacy risks, which could affect long-term acceptability. To help mitigate this, a prior qualitative study was conducted with the same Korail community to explore participants’ understanding, perspectives, and willingness to share digital data [87]. Insights from this study informed the design of our consent process and data collection strategy. Nevertheless, residual concerns about privacy remain a possible barrier. We will therefore monitor such issues throughout this study and systematically document dropout or data loss attributable to these barriers.

### Conclusions

This study represents a step forward in understanding how DP and ML can be used to predict relapse in individuals with serious mental disorders living in low-resource settings. By combining passive smartphone data with clinical assessments, we aim to build a predictive model that can identify the EWS of relapse, allowing for timely interventions.

While there are challenges associated with the use of DP, such as data privacy and the interpretation of behavioral markers, the potential benefits for early detection and intervention in mental health are substantial. The findings from this study will not only contribute to the growing body of research on DP but also offer practical solutions for addressing the mental health treatment gap in underserved populations. Ultimately, this research could help improve the quality of mental health care in LMIC settings and impoverished communities and provide a model for scalable, technology-driven interventions in other low-resource environments. This research has the potential to inform both local and global strategies for improving access to mental health care in low-resource settings.

## Abbreviations

BSI: Brief Symptom Inventory
DP: digital phenotyping
*DSM-5*: *Diagnostic and Statistical Manual of Mental Disorders, Fifth Edition*
EDF: effective degrees of freedom
EPP: events per predictor
EWS: early warning sign
GAF: Global Assessment of Functioning
GSI: Global Severity Index
HIC: high-income country
LASSO: least absolute shrinkage and selection operator
LMIC: low- and middle-income country
MDD: major depressive disorder
ML: machine learning
NIHR: National Institute for Health and Care Research
NLP: natural language processing
PANSS: Positive and Negative Syndrome Scale
PIS: participant information sheet
PTSD: posttraumatic stress disorder
SHAP: Shapley Additive Explanations
SMI: serious mental illness
TRANSFORM: Transforming Access to Care for Serious Mental Disorders in Slums
WHOQOL-BREF: World Health Organization Quality of Life-Brief
